# Maximal Oxygen Uptake Adjusted for Skeletal Muscle Mass in Competitive Speed-Power and Endurance Male Athletes: Changes in a One-Year Training Cycle

**DOI:** 10.3390/ijerph17176226

**Published:** 2020-08-27

**Authors:** Jacek Trinschek, Jacek Zieliński, Krzysztof Kusy

**Affiliations:** Department of Athletics, Strength and Conditioning, Faculty of Sport Sciences, Poznan University of Physical Education, ul. Królowej Jadwigi 27/39, 61-871 Poznań, Poland; jactri@wp.pl (J.T.); jacekzielinski@wp.pl (J.Z.)

**Keywords:** maximum aerobic capacity, total weight, lean body mass, fat mass, DXA method

## Abstract

We compared the changes in maximum oxygen uptake ($\overset{\cdot }{V}O_{2}$max) calculated per skeletal muscle mass (SMM) with conventional $\overset{\cdot }{V}O_{2}$max measures in a 1-year training cycle. We hypothesized that the pattern of changes would differ between SMM-adjusted and absolute or weight-adjusted values, and the differences between groups of distinct training specialization and status will depend on the measure used. Twelve sprinters (24.7 ± 3.3 years), 10 endurance runners (25.3 ± 5.3 years), and 10 recreationally trained controls (29 ± 4.5 years) performed a treadmill test until exhaustion to determine $\overset{\cdot }{V}O_{2}$max. Their SMM was estimated based on the dual X-ray absorptiometry method and a regression equation. The significance of differences was assessed using analysis of variance (*p* ≤ 0.05). The pattern of the longitudinal change was not different between $\overset{\cdot }{V}O_{2}$max/SMM and standard measures. Also, the significance of differences between sprinters and endurance athletes remained similar regardless of the $\overset{\cdot }{V}O_{2}$max measure. Sprinters and controls had similar absolute (~4.3 L·min^−1^) and total weight-adjusted (~52 vs. ~56 mL·min^−1^·kg) $\overset{\cdot }{V}O_{2}$max, but they significantly differed in SMM-adjusted $\overset{\cdot }{V}O_{2}$max (~110 vs. ~130 mL·min^−1^·kg SMM^−1^). In summary, SMM-adjusted $\overset{\cdot }{V}O_{2}$max is not more useful than standard measures to track longitudinal changes in competitive athletes. However, it allows to better distinguish between groups or individuals differing in training status. The results of our study are limited to male athletes.

## 1. Introduction

Maximal oxygen uptake ($\overset{\cdot }{V}O_{2}$max) is a widely used indicator of human aerobic capacity defined as the maximum rate of oxygen consumption. Conventionally, $\overset{\cdot }{V}O_{2}$max is expressed as an absolute rate of oxygen uptake per unit of time (mL·min^−1^) or as a weight-adjusted rate (mL·min^−1^·kg^−1^) [1,2,3,4]. The latter is a standard measure in athletes of various sports disciplines. Skeletal muscle mass (SMM) is the largest component of the adipose tissue-free body mass in humans [5], essential for athletic performance. Despite many differences in training and competition specificity, available research indicates that SMM content in athletes ranges from 40% to 48% of total body mass [6,7,8,9,10,11].

The body of literature on $\overset{\cdot }{V}O_{2}$max in competitive athletes in the context of SMM is very scarce (unlike the relationships with total body mass). This can be due to problems with accurate SMM estimation. Of particular interest are, therefore, studies where authors used most advanced methods, e.g., magnetic resonance imaging or dual-energy X-ray absorptiometry (DXA) to estimate SMM. Proctor & Joyner [11] demonstrated that reduced aerobic capacity per kilogram of appendicular SMM in highly trained older men and women contributed to reduced whole body $\overset{\cdot }{V}O_{2}$max. Sanada et al. [12] revealed that absolute peak $\overset{\cdot }{V}O_{2}$ was closely associated with total and regional SMM regardless of the whole body or fat-free mass. Similarly, Beekley et al. [13] indicated a strong relationship between SMM (kg) and absolute $\overset{\cdot }{V}O_{2}$max (L·min^−1^) in high-performance athletes. However, they also noticed that above a certain SMM level (~45 kg), the relationship between $\overset{\cdot }{V}O_{2}$ uptake and SMM was weakening and aerobic abilities of athletes reached a “plateau”.

It is suggested that in highly trained athletes, not only standard measures of $\overset{\cdot }{V}O_{2}$ (per kg of total body mass) but also SMM-adjusted $\overset{\cdot }{V}O_{2}$max, called “aerobic muscle quality”, should be taken into account to obtain more accurate and reliable information on the changes in the training status [13]. To our best knowledge, there are no scientific reports that have compared the changes in SMM-adjusted $\overset{\cdot }{V}O_{2}$max in high-performance athletes of different specializations over a long period. This study aimed to evaluate the changes in SMM-adjusted $\overset{\cdot }{V}O_{2}$max in competitive highly trained speed-power and endurance athletes in a 1-year training cycle. We hypothesized that (i) the profile of changes in $\overset{\cdot }{V}O_{2}$max per kg SMM would differ from that per kg total body mass and (ii) the size of the differences in $\overset{\cdot }{V}O_{2}$max between speed-power, endurance, and amateur male athletes would depend on the measure of $\overset{\cdot }{V}O_{2}$max used (SMM- vs. total weight-adjusted).

## 2. Materials and Methods

### 2.1. Subjects

The study included 22 highly trained male athletes divided into two groups differing in sport specialization. Sprinters (*n* = 12) specialized in the distances of 100 and 200 m, were 24.7 ± 3.3 (range 21–31) years old with a training history of 7.42 ± 2.5 years. Endurance athletes were long-distance runners and triathletes (*n* = 10) aged 25.3 ± 5.3 (range 15–35) years with a competitive sport history of 8.0 ± 2.4 years. Some athletes were members of the Polish national team. The control group consisted of 10 healthy recreationally active men aged 29 ± 4.5 (range 23–35) years without previous and current professional sports experience, representing the model of regular but not competitive physical activity. The controls were invited to participate in the study through announcements in local mass media. The project was approved by the Ethics Committee at the Poznan University of Medical Sciences (decision No 1252/18 issued on 6 December 2018) and has been performed according to the ethical standards laid down in the Declaration of Helsinki. The participants were fully informed of the purpose and risks of the study and gave their written consent to participate. Basic characteristics of the participants at the start of the study are presented in Table 1. Controls were older than athletes. Sprinters were taller and had higher relative skeletal muscle mass index than endurance athletes and controls.

### 2.2. Study Design

A repeated-measures design was used to follow the changes in $\overset{\cdot }{V}O_{2}$max and body composition across a 1-year training cycle. We aimed to find patterns of the longitudinal change and between-group differences depending on the $\overset{\cdot }{V}O_{2}$max measure, i.e., absolute, weight-, LBM-, and SMM-adjusted values. All measurements were repeated four times in the following training phases of the annual training cycle: (1) beginning of the general preparation period, (2) beginning of the specific preparation period, (3) beginning of the pre-competition period, and (4) beginning of the competition period. Training units and workloads used in the training process were strictly planned by the national team coaches. The 12-week general preparation aimed to develop physiological foundation for performance. Training volume was high and the intensity was low but slowly increasing (the number of training sessions in triathletes, long-distance runners, and sprinters was 181, 122, and 80, respectively). During the specific preparation period, also lasting 12 weeks, training volume decreased, whereas the intensity increased substantially (the number of training sessions: 132, 96, and 61, respectively). In the pre-competition period (10 weeks), training volume further decreased and the intensity increased (the number of training sessions: 179, 120, and 87, respectively). The competition period was characterized by reduced training volume and emphasis was placed on increasing intensity and quality of work to achieve peak performance before upcoming competitions. Sprinters were examined three times, i.e., they did not perform the exercise test until exhaustion in the competition phase to avoid any adverse effect on sprint ability. The control group did not periodize their training during the year analyzed. During the whole study period, they did workouts three to seven times a week at relatively constant training volume and intensity.

### 2.3. Methodology

Participants were recommended to avoid high-intensity and long-duration training sessions 24–48 h before each examination. All tests were conducted at the Human Movement Laboratory “LaBthletics” of the Poznan University of Physical Education. The measurements were performed in the morning, 2 h after a light breakfast (bread and butter, water, without coffee or tea). Before each exercise test, body composition was assessed. Then, subjects performed an incremental treadmill test until exhaustion. During all examinations, the ambient temperature was kept at 20–21 °C.

#### 2.3.1. Body Composition and Skeletal Muscle Mass

Weight and height were measured using the SECA 285 measuring station (SECA GmbH, Hamburg, Germany) with an accuracy of 0.05 kg and 1 mm, respectively. To evaluate body composition, the DXA method (Lunar Prodigy device, GE Healthcare, Chicago, IL, USA) was used. Before each measurement session, the device was calibrated using a phantom, according to the manufacturer guidelines. During the examination, subjects only wore their underwear without jewelry or other metal objects, to minimize measurement error. All DXA scans were performed and analyzed by the same trained technician using enCORE 16 SP1 software (GE Healthcare, Chicago, IL, USA). All measurements were done following the standardized protocol proposed by Nana et al. [14] and manufacturer’s instructions. Three main components of the total-body model were measured: lean body mass (LBM), fat mass, and bone mineral content (the latter not analyzed in this study). In the literature, the DXA technical errors of measurement (expressed as intra-assay coefficients of variation or %CV) have been reported to be 0.1% for total body mass, 0.4% for LBM, 1.9% for fat mass, and 0.7% for BMC (21). In our laboratory, %CV values in young athletic individuals aged 23 ± 2.1 years were 0.2%, 0.4%, 1.0%, and 0.5%, respectively. Also, we calculated %CV for appendicular lean soft tissue (ALST; the sum of upper and lower limb LBM) and obtained a value of 0.8%. The regression model proposed by Kim et al. [5] was used to calculate SMM (kg) = 1.13ALST − 0.02Age + 0.61Sex + 0.97, where 0 and 1 denoted women or men, respectively. Also, the relative skeletal muscle mass index was calculated according to the formula: RSMI = ALST/Height^2^ (kg·m^−2^).

#### 2.3.2. Maximum Oxygen Uptake

All athletes underwent incremental running tests (h/p Cosmos Pulsar treadmill, Sports & Medical GmbH, Nussdorf-Traunstein, Germany) to determine $\overset{\cdot }{V}O_{2}$max. The initial speed was set at 4 km·h^−1^ and after 3 min increased to 8 km·h^−1^. After that point, the speed of the moving strip was progressively increasing by 2 km·h^−1^ every 3 min until voluntary exhaustion. Main cardiorespiratory variables (minute ventilation, $\overset{\cdot }{V}$E; oxygen uptake, $\overset{\cdot }{V}O_{2}$; carbon dioxide output, $\overset{\cdot }{V}CO_{2}$) were measured constantly (breath by breath) using the MetaLyzer 3B ergospirometer and analyzed using the MetaSoft Studio 5.1.0 software package (Cortex Biophysik GmbH, Leipzig, Germany). Before each test, the system was calibrated according to the manufacturer’s instructions. Maximal oxygen uptake was considered achieved if at least three of the following criteria were met: (i) a plateau in $\overset{\cdot }{V}O_{2}$ despite an increase in speed and minute ventilation; (ii) blood lactate concentration ≥ 9 mmol·L^−1^; (iii) respiratory exchange ratio ≥ 1.10; and (iv) heart rate ≥ 95% of the age-predicted maximum heart rate [15]. Heart rate was measured continuously with the Polar Bluetooth Smart H6 monitor (Polar Electro Oy, Kempele, Finland).

#### 2.3.3. Statistical Analysis

Data were presented as means and standard deviations (SD), and confidence intervals of the mean (95% CI). The Shapiro–Wilk test was used to check the data for normality of distribution. The assumption on sphericity was tested using the Mauchley’s test, verifying if variances of certain variables were identical and equal to respective co-variances. The one-way analysis of variance (ANOVA) with repeated measures was used to compare the change between three (sprinters) or four (endurance athletes and controls) examinations across the annual training cycle. The one-way ANOVA was used to compare differences between the groups at each single training phase. The post hoc Scheffe’s test was applied to indicate between which particular examinations or groups there were significant differences. The effect size for ANOVA was expressed as η^2^ and defined as small (0.01), medium (0.06), or large (0.14). The statistical significance was set at *p* < 0.05. All analyses were performed using the Statistica 13.0 software package (Tibco Software Inc., Palo Alto, CA, USA).

## 3. Results

### 3.1. Body Composition

Sprinters had significantly higher total body mass than endurance athletes in three training periods (general, specific and pre-competition) (Table A1). In sprinters, total mass increased from general to specific and pre-competition phases, whereas no significant longitudinal changes were revealed in endurance athletes and controls.

**Figure 1:**
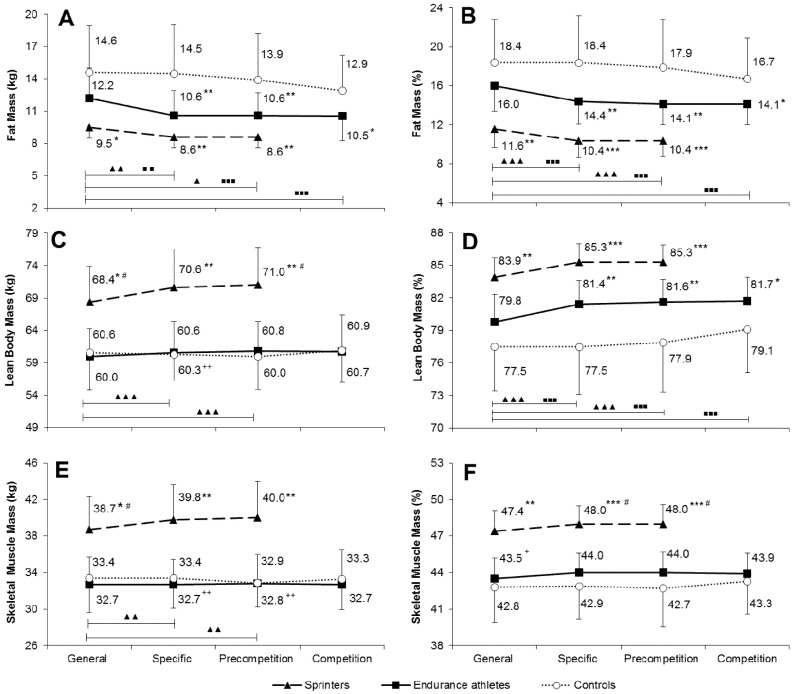
Changes in body composition expressed in absolute and percentage values between consecutive phases of the annual training cycle in athletic groups and controls. Panels (**A**,**B**)—fat mass; Panels (**C**,**D**)—lean body mass; Panels (**E**,**F**)—skeletal muscle mass. ^▲^ *p* < 0.05, ^▲▲^ *p* < 0.01, ^▲▲▲^ *p* < 0.001—significantly different from the general preparation phase in sprinters; ^■■^ *p* < 0.01, ^■■■^ *p* < 0.001—significantly different from the general preparation phase in endurance athletes; ^+^ *p* < 0.05, ^++^ *p* < 0.01—significantly different from sprinters at the same training phase; ^#^ *p* < 0.01—significantly different from endurance athletes at the same training phase; * *p* < 0.05, ** *p* < 0.01, *** *p* < 0.001—significantly different from controls at the same training phase.

Absolute and percentage fat mass was similar in sprinters and endurance athletes in all examinations, although slightly lower values were noted in sprinters (insignificant differences) (Figure 1A,B; Table A1). Both sprint and endurance groups had significantly lower absolute and percentage fat mass than controls in almost all examinations, except for the general phase (a non-significant difference between endurance athletes and controls). In sprinters and endurance athletes, absolute and percentage fat mass was significantly higher in the general phase than in the subsequent training phases. No significant change was detected in controls, even though there was a certain trend towards lower values in the competition phase, however, accompanied by large standard deviation.

Sprinters had significantly higher absolute LBM than endurance athletes and controls in the general, specific, and pre-competition phases (Figure 1C; Table A1). Endurance athletes had higher percentage LBM than controls in all training phases, except for the general phase (Figure 1D). Absolute LBM in sprinters and percentage LBM in both sprinters and endurance runners significantly increased between the general and the subsequent training phases (Figure 1C,D). No significant change in LBM was shown in the control group, in spite of slightly increasing percentage values between third and fourth examination (Figure 1D).

Sprinters had significantly higher both absolute and percentage SMM than endurance athletes and controls in all training phases (Figure 1E,F; Table A1). There were no significant differences in SMM between endurance athletes and controls. In sprinters (but not endurance athletes and controls), absolute SMM significantly increased from the general to the specific and pre-competition phases (Figure 1E). During the annual training cycle, there was no significant change in percentage SMM in any of the three groups (Figure 1F).

### 3.2. Maximal Oxygen Uptake

**Figure 2:**
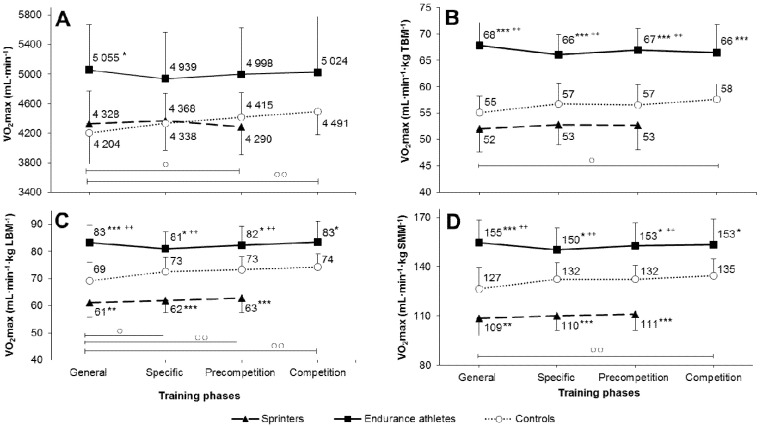
Changes in maximal oxygen uptake ($\overset{\cdot }{V}O_{2}$max) between consecutive phases of the annual training cycle in athletes and controls. (**A**)—absolute values; Panel (**B**)—calculated per weight or kilogram of total body mass (TBM); Panel (**C**)—calculated per kilogram of lean body mass (LBM); Panel (**D**)—calculated per kilogram of skeletal muscle mass (SMM.) ^○^ *p* < 0.05, ^○○^ *p* < 0.01—significantly different from the general preparation phase in the control group; ^++^ *p* < 0.01—significantly different from sprinters at the same training phase; ^#^ *p* < 0.01—significantly different from endurance athletes at the same training phase; * *p* < 0.05, ** *p* < 0.01, *** *p* < 0.001—significantly different from controls at the same training phase.

Across the annual training cycle, a significant increase in all $\overset{\cdot }{V}O_{2}$max indicators (absolute, per total body mass, per LBM, or per SMM) was only observed in controls between the general and pre-competition or competition phase. In sprinters and endurance runners, none of the $\overset{\cdot }{V}O_{2}$max measures changed significantly (Figure 2A–D; Table A2).

Depending on the $\overset{\cdot }{V}O_{2}$max indicator used, the significance of the difference between speed-power, endurance, and amateur athletes varied. For absolute $\overset{\cdot }{V}O_{2}$max (mL·min^−1^), the only significant difference was between endurance athletes and controls in the general preparation phase (Figure 2A, Table A2). For weight-adjusted $\overset{\cdot }{V}O_{2}$max, more pronounced differences were observed, i.e., endurance athletes significantly differed from speed-power and control groups in all training periods (Figure 2B; Table A2), however, sprinters and controls were not significantly different. Finally, when $\overset{\cdot }{V}O_{2}$max was adjusted for LBM and SMM, there emerged significant differences between sprinters and controls in addition to previous differences for weight-adjusted $\overset{\cdot }{V}O_{2}$max between endurance athletes and the other two groups. Consequently, the control group had higher LBM- and SMM-adjusted $\overset{\cdot }{V}O_{2}$max than sprinters in all training phases (Figure 2C,D; Table A2).

## 4. Discussion

To our knowledge, this is the first study to analyze the changes in $\overset{\cdot }{V}O_{2}$max calculated per SMM across an annual training cycle in competitive athletes of different sports specializations. The major findings are that (i) the profile of change in SMM-adjusted $\overset{\cdot }{V}O_{2}$max in a 1-year training cycle is not different from the change in weight-adjusted $\overset{\cdot }{V}O_{2}$max and (ii) the between-group differences depend on the $\overset{\cdot }{V}O_{2}$max measure used, as shown by significant differences between sprinters and controls that emerged when SMM- or LBM-adjusted $\overset{\cdot }{V}O_{2}$max values were used.

### 4.1. Changes in $\overset{\cdot }{V}O_{2}$max between Training Phases

There is scarce research on $\overset{\cdot }{V}O_{2}$max expressed as relative values per kg of SMM [11,12]. Nevertheless, there were reasons to believe that the profile of the changes in SMM-adjusted $\overset{\cdot }{V}O_{2}$max across a 1-year training cycle would be different from that expressed as absolute and weight-adjusted values. However, this hypothesis has not been confirmed. In sprint- and endurance -trained athletes and controls, the profiles of change across training phases were very similar regardless of $\overset{\cdot }{V}O_{2}$max measure.

In endurance athletes, high $\overset{\cdot }{V}O_{2}$max is regarded as one of the necessary (although not sufficient) factors determining high endurance performance [16,17]. In response to years of intense training (apart from innate aptitudes), the level of $\overset{\cdot }{V}O_{2}$max is usually maximized and the observed seasonal changes can be negligible. Due to optimally high $\overset{\cdot }{V}O_{2}$max levels, endurance athletes are focused on other factors determining performance such as exercise response at lactate (anaerobic) threshold or exercise efficiency, e.g., “running economy” meaning the oxygen cost at a given running speed [16,18,19]. It seems that no indicator of maximal aerobic capacity, whether it be a weight- or SMM-adjusted one, is suitably sensitive to track training adaptations in highly trained athletes. On the other hand, the effect of body composition on endurance performance is still valid. For example, in male trained trial runners (age 36.1 ± 6.5 years), $\overset{\cdot }{V}O_{2}$max and fat mass percent were the two best predictors of race time among other physiological and body composition variables, explaining ~84% of the total variance in a multiple regression model [20]. Even if not considered in terms of cause and effect, the changes in body composition across training phases just accompany improvements in aerobic capacity and endurance performance as related physiological adaptations [21].

Available research indicates that body composition and its variations have a significant impact on $\overset{\cdot }{V}O_{2}$max. As mentioned in the introduction, absolute SMM in athletes is strongly directly proportional to absolute oxygen uptake (up to the suggested limit of ~45 kg of SMM). In highly trained male rowers (20 ± 2 years old), it was predicted based on a regression model that an increase in fat free mass by 1 kg should result in the gain in $\overset{\cdot }{V}O_{2}$max by 0.16 L·min^−1^ [22]. Also, it is known that body fat strongly negatively correlates with $\overset{\cdot }{V}O_{2}$max [13,23]. It can be assumed that body composition does matter in achieving high levels of aerobic capacity. However, our results showed that, despite reductions in absolute and percentage fat mass in endurance athletes across the training phases, there was no positive effect on $\overset{\cdot }{V}O_{2}$max. The likely explanation is that some determinants of $\overset{\cdot }{V}O_{2}$max, e.g., muscle adaptation to endurance training such as mitochondrial enzyme levels, capillary density or other central and peripheral factors [17,24,25], were maximized and could not be substantially improved in this highly trained group. Moreover, particular effects and relationships seem to depend on age, sex, training status, and athletic profile.

In controls, unlike in athletes, we observed significant changes in $\overset{\cdot }{V}O_{2}$max indicators between consecutive examinations, despite no significant change in body components. Zwaard et al. [4] suggested that in amateurs adaptive changes such as capillaries or the type of muscle fibers were not as one-sided directed as in endurance- or sprint-trained professional athletes. Central and peripheral adaptations supporting $\overset{\cdot }{V}O_{2}$max were not maximized in recreationally active individuals, thus our control group could have more strongly responded to training stimuli, even if their training loads were milder than in competitive athletes, because of their relatively low baseline level of $\overset{\cdot }{V}O_{2}$max (compared to the other two groups) at the start the annual cycle under consideration.

In sprinters, the expected lack of significant changes in $\overset{\cdot }{V}O_{2}$max between consecutive training phases (despite desired changes in body composition, i.e., fat mass reduction) results from their specific training and performance requirements. Sprint is an all-out high-intensity exercise and the distance covered during competition (including acceleration, achieving maximal velocity, and deceleration) lasts for up to several seconds [26]. It is recommended that elite sprinters should primarily focus on increasing their relative muscle power production using ballistic exercises to maximize speed performance [27]. It is clear that $\overset{\cdot }{V}O_{2}$max is not crucial for sprint performance. On the other hand, at the early stage of the annual training cycle (the general preparation phase), sprinters’ workouts include a certain amount of aerobic exercise to reach an optimal level of aerobic capacity. This allows speed-power athletes to better tolerate training loads necessary for speed and speed endurance development [28]. Despite the significant decrease in body fat in sprinters, there was no positive effect on their $\overset{\cdot }{V}O_{2}$max. This may be associated with the simultaneous increase in SMM, the large amount of which is typical of sprinters. Our sprinters have approached the “upper limit” of SMM (~40 kg vs. the ~45 kg proposed by Beekley [13]), beyond which $\overset{\cdot }{V}O_{2}$max is plateauing or even decreasing. More importantly, skeletal muscles in speed-power athletes are characterized by a relatively low capillary density and mitochondrial density, resulting in lower O_2_ extraction from the blood by contracting muscles and, consequently, in lower $\overset{\cdot }{V}O_{2}$max [4,29]. This athletic group focuses on training supporting anaerobic metabolic systems that are the main energy source for muscle activity during sprint running [30,31]. Such training results in a low content of aerobic enzymes in skeletal muscle [32,33].

### 4.2. Between-Group Differences in $\overset{\cdot }{V}O_{2}$max

In a few previous studies on the relationship between SMM and $\overset{\cdot }{V}O_{2}$max, it was suggested that $\overset{\cdot }{V}O_{2}$max normalized to skeletal muscle mass might be a more relevant index than simply weight-adjusted $\overset{\cdot }{V}O_{2}$max in the evaluation of aerobic power [11,12]. Moreover, Beekley et al. [13], developed the term “aerobic muscle quality”, meaning the amount of oxygen consumed per 1 kg of SMM, to make better comparisons of $\overset{\cdot }{V}O_{2}$max between individuals of varying fat and total body mass or representing different sports.

In our study, significant differences in SMM- or LBM-adjusted $\overset{\cdot }{V}O_{2}$max were revealed between sprinters and controls, contrary to non-significant differences in absolute and weight-adjusted values. This can be affected by several factors. First, as other authors have suggested, adjustment of $\overset{\cdot }{V}O_{2}$max for fat-free mass or skeletal muscle mass is out the influence of adipose tissue [10]. In our sprinters, LBM and SMM significantly increased across the annual cycle, while these body components remained unchanged in controls. This caused significant differences between sprinters and controls in both SMM- and LBM-adjusted $\overset{\cdot }{V}O_{2}$max, while there was no significant change in absolute and weight-adjusted $\overset{\cdot }{V}O_{2}$max. Second, the control group consisted of recreationally active men whose physical activity was endurance-oriented. Endurance training modifies central (pulmonary diffusing capacity, maximal cardiac output, the oxygen-carrying capacity of the blood) and peripheral (skeletal muscle characteristics) factors affecting $\overset{\cdot }{V}O_{2}$max [16,18]. For example, skeletal muscles that undergo endurance training oxidize fat at a higher rate (thus sparing muscle glycogen and blood glucose) and contribute to the decrease in lactate production during exercise. Besides, more muscle mitochondria allow more oxygen to be extracted from the blood by contracting muscles [16,18]. Such typical muscle adaptations (occurring in endurance- but not sprint-trained individuals) may explain significant differences in SMM-adjusted $\overset{\cdot }{V}O_{2}$max that emerged between sprinters and controls, even though they were not detectable when standard $\overset{\cdot }{V}O_{2}$max measures were used.

In our participants, the percentage of LBM ranged between 78% and 85% of total body mass, whereas the percentage of SMM was between 43% and 48% (Figure 1). However, despite such a sizeable quantitative difference between these body components as regards their contribution to the total weight, adjusting $\overset{\cdot }{V}O_{2}$max for SMM only slightly (by mere ~2%) deepened the differences between sprinters and controls compared to LBM-adjusted values (Figure 2). In practical terms, it can be, therefore, assumed that LBM- and SMM-adjusted $\overset{\cdot }{V}O_{2}$max provide virtually the same information. Thus, calculating the SMM-adjusted $\overset{\cdot }{V}O_{2}$max to compare groups of different training status seems to be unnecessary. Using LBM, which itself contains about 55–57% SMM, to more precisely express the $\overset{\cdot }{V}O_{2}$max level, can be quite sufficient and easier.

## 5. Conclusions

In summary, our research has proven that in endurance- and sprint-trained competitive athletes and recreationally active individuals the profiles of 1-year changes in SMM-adjusted vs. weight-adjusted $\overset{\cdot }{V}O_{2}$max are not different. However, adjusting $\overset{\cdot }{V}O_{2}$max for LBM or SMM can uncover significant differences in maximal aerobic capacity between groups of different training specialization and status. In high-performance athletes, the use of the LBM- or SMM-adjusted $\overset{\cdot }{V}O_{2}$max as an index of “aerobic muscle quality” to track the changes in maximum aerobic capacity across consecutive training phases seems to be unjustified. In competitive athletes, the monitoring and control of maximum aerobic capacity across an annual training cycle can be successfully carried out using conventional (absolute and weight-adjusted) $\overset{\cdot }{V}O_{2}$max measures. Admittedly, the LBM- or SMM-adjusted $\overset{\cdot }{V}O_{2}$max can be useful as a tool to more precisely distinguish between groups or individuals differing in muscle adaptation to maximum oxygen uptake. It also seems that LBM- and SMM-adjusted $\overset{\cdot }{V}O_{2}$max measures provide equivalent information about maximum aerobic capacity. Finally, the limitation of our study is the participation of only male athletes, thus further research is needed to explore analogous patterns of change in different $\overset{\cdot }{V}O_{2}$max measures in female athletes.

## Figures and Tables

**Table 1:** Basic characteristics of the athletic groups and controls.

	Sprint	Endurance	Controls	ANOVA *p*-Value	Effect Size η^2^
Age (yars)	24.7 ± 3.3 (22.1–26.2) *	25.3 ± 5.3 (22.3–28.2) *	29 ± 4.5 (26–32)	<0.001	0.22
Sports history (years)	7.4 ± 2.5 (5.8–9.0)	8.0 ± 2.4 (6.3–9.7)	--	0.120	0.30
Height (cm)	185.8 ± 5.0 (182.1–188.2) *	181.6 ± 6.1 (178.2–185)	178.1 ± 5.6 (174.3–181.9)	0.029	0.33
BMI (kg·m^−2^)	23.6 ± 1.0 (22.8–24.3)	22.8 ± 1.9 (21.8–23.9)	24.8 ± 2.0 (23.4–26.1)	0.080	0.24
RSMI (kg)	9.6 ± 0.6 ^#^ (9.1–10.0) ^#^	8.5 ± 0.6 (8.1–8.9)	9.0 ± 0.6 (8.6–9.4)	0.007	0.42

Values are expressed as mean ± SD (95% CI). Abbreviations: BMI = body mass index; RSMI = relative skeletal muscle mass index. * *p* < 0.05—significantly different from the control group; ^#^ *p* < 0.01—significantly different from endurance athletes.

## Appendix A

**Table A1:** Changes in body composition between consecutive phases of the annual training cycle in athletic groups and controls.

	Training Phases				ANOVA Btw. Phases	Effect Size η^2^
	General	Specific	Pre-Competition	Competition		
**Total Mass (kg)**						
Sprint	81.6 ± 5.9	82.8 ± 6.3 ^▲#^	83.3 ± 6.3 ^▲▲▲^	-	<0.001	0.39
Endurance	75.4 ± 7.4 ^+^	74.5 ± 6.5	74.6 ± 6.2 ^+^	74.4 ± 6.7	0.138	0.12
Controls	78.4 ± 5.8	78.0 ± 6.2	77.1 ± 6.7	77.0 ± 5.6	0.165	0.17
ANOVA btw. Groups	0.033	0.006	0.019	0.179		
Effect Size η^2^	0.32	0.43	0.36	0.19		
**Fat Mass (kg)**						
Sprinters	9.5 ± 1.5 *	8.6 ± 1.4 ^▲▲^**	8.6 ± 1.4 ^▲^**	-	0.002	0.32
Endurance	12.2 ± 2.8	10.6 ± 2.3 ^■■^**	10.6 ± 2.1 ^■■■^**	10.54 ± 2.3 ^■■■^*	<0.001	0.46
Controls	14.6 ± 4.3	14.5 ± 4.5	13.9 ± 4.3	12.9 ± 3.3	0.109	0.20
ANOVA btw. Groups	0.011	<0.001	0.001	0.022		
Effect Size η^2^	0.40	0.55	0.52	0.46		
**Fat Mass (%)**						
Sprinters	11.6 ± 1.9 **	10.38 ± 1.7 ^▲▲▲^***	10.36 ± 1.6 ^▲▲▲^***	-	<0.001	0.43
Endurance	16.00 ± 2.6	14.4 ± 2.3 ^■■■^**	14.1 ± 2.1 ^■■■^**	14.1 ± 2.1 ^■■■^*	<0.001	0.46
Controls	18.4 ± 4.4	18.4 ± 4.8	17.9 ± 4.9	16.7 ± 4.2	0.149	0.18
ANOVA btw. Groups	0.002	<0.001	<0.001	0.039		
Effect Size η^2^	0.51	0.64	0.65	0.39		
**Lean Body Mass (kg)**						
Sprinters	68.4 ± 5.5 ^#^*	70.6 ± 5.9 ^▲▲▲^**	71.0 ± 5.8 ^▲▲▲#^**	-	<0.001	0.72
Endurance	60.0 ± 5.2	60.6 ± 4.8 ^++^	60.8 ± 4.6	60.7 ± 4.7	0.140	0.12
Controls	60.6 ± 3.6	60.3 ± 4.1	60.0 ± 5.1	60.9 ± 5.5	0.511	0.08
ANOVA btw. Groups	0.002	<0.001	<0.001	0.61		
Effect Size η^2^	0.49	0.60	0.55	0.03		
**Lean Body Mass (%)**						
Sprinters	83.9 ± 1.8 **	85.3 ± 1.7 ^▲▲▲^***	85.3 ± 1.6 ^▲▲▲^***	-	<0.001	0.48
Endurance	79. 8 ± 2.5	81.4 ± 2.2 ^■■■^**	81.6 ± 2.1 ^■■■^**	81.7 ± 2.2 ^■■■^*	<0.001	0.47
Controls	77.5 ± 4.1	77.5 ± 4.4	77.9 ± 4.6	79.1 ± 4.0	0.165	0.17
ANOVA btw. groups	0.001	<0.001	<0.001	0.036		
Effect size η^2^	0.52	0.66	0.67	0.40		
**Skeletal Muscle Mass (kg)**						
Sprinters	38.7 ± 3.6 ^#^*	39.8 ± 3.8 ^▲▲^**	40.0 ± 4.0 ^▲▲^**	-	<0.001	0.37
Endurance	32.7 ± 3.1	32.7 ± 2.6 ^++^	32.8 ± 2.6 ^++^	32.7 ± 2.8	0.961	0.01
Controls	33.4 ± 2.3	33.4 ± 2.1	32.9 ± 3.1	33.3 ± 3.2	0.561	0.07
ANOVA btw. Groups	0.002	<0.001	<0.001	0.503		
Effect Size η^2^	0.51	0.64	0.60	0.05		
**Skeletal Muscle Mass (%)**						
Sprinters	47.4 ± 1.7 **	48.0 ± 1.5 ^#^***	48.0 ± 1.6 ^#^***	-	0.198	0.10
Endurance	43.5 ± 1.7 ^+^	44.0 ± 1.6	44.0 ± 1.7	43.9 ± 1.7	0.108	0.13
Controls	42.8 ± 2.9	42.9 ± 2.7	42.7 ± 3.2	43.3 ± 2.7	0.631	0.06
ANOVA btw. Groups	0.001	<0.001	<0.001	0.036		
Effect Size η^2^	0.52	0.62	0.70	0.24		

^▲^*p* < 0.05, ^▲▲^ *p* < 0.01, ^▲▲▲^ *p* < 0.001—significantly different from the general preparation phase in a sprinters group; ^■■^ *p* < 0.01, ^■■■^ *p* < 0.001—significantly different from the general preparation phase in endurance group; ^+^ *p* < 0.05, ^++^ *p* < 0.01—significantly different from sprinters at the same training phase, ^#^ *p* < 0.01—significantly different from endurance athletes at the same training phase, * *p* < 0.05, ** *p* < 0.01, *** *p* < 0.001—significantly different from controls at the same training phase.

**Table A2:** Changes in maximal oxygen uptake (absolute and relative values) between consecutive phases of the annual training cycle in athletic groups and controls.

	Training Phases				ANOVA Btw. Phases	Effect Size η^2^
	General	Specific	Pre-Competition	Competition		
**$\overset{\cdot }{V}O_{2}$max (mL·min^−1^)**						
Sprint	4328 ± 438	4368 ± 370	4290 ± 381	--	0.268	0.08
Endurance	5055 ± 615 *	4939 ± 628.15	4998 ± 628	5024 ± 753	0.628	0.04
Controls	4204 ± 419	4338 ± 365	4415 ± 335 ^○^	4491 ± 315 ^○○^	0.001	0.45
ANOVA btw. Groups	0.022	0.232	0.077	0.487		
Effect Size η^2^	0.34	0.15	0.25	0.06		
**$\overset{\cdot }{V}O_{2}$max (mL·min^−1^·kg TBM^−1^)**						
Sprinters	52.1 ± 4.5	52.8 ± 3.7	52.7 ± 4.6		0.406	0.05
Endurance	67.9 ± 4.3 ^++^***	66.1 ± 3.9 ^++^***	66.9 ± 4.1 ^++^***	66.5 ± 5.3 ***	0.467	0.06
Controls	54.6 ± 3.4	56.4 ± 3.9	56.7 ± 3.7	57.4 ± 2.8 ^○^	0.033	0.27
ANOVA btw. Groups	>0.001	>0.001	>0.001	>0.001		
Effect Size η^2^	0.86	0.78	0.76	0.78		
**$\overset{\cdot }{V}O_{2}$max (mL·min^−1^·kg LBM^−1^)**						
Sprinters	61.1 ± 5.4 **	62.0 ± 4.5 ***	62.7 ± 5.4 ***	--	0.077	0.15
Endurance	83.2 ± 6.4 ^++^***	81.0 ± 6.1 ^++^*	82.4 ± 6.9 ^++^*	83.4 ± 7.8 *	0.321	0.08
Controls	69.2 ± 6.7	72.5 ± 5.2 ^○^	73.3 ± 4.7 ^○○^	74.2 ± 4.0 ^○○^	0.001	0.47
ANOVA btw. Groups	>0.001	>0.001	>0.001	0.011		
Effect Size η^2^	0.83	0.81	0.79	0.53		
**$\overset{\cdot }{V}O_{2}$max (mL·min^−1^·kg SMM^−1^)**						
Sprinters	108.6 ± 10.6 **	110.2 ± 9.2 ***	111.2 ± 10.1 ***	--	0.306	0.07
Endurance	154.8 ± 13.4 ^++^***	150.5 ± 13.1 ^++^*	152.7 ± 14.0 ^++^*	153.4 ± 15.7 *	0.404	0.07
Controls	126.7 ± 12.9	132.4 ± 9.9	132.4 ± 8.4	134.7 ± 10.0 ^○^	0.005	0.37
ANOVA btw. Groups	>0.001	>0.001	>0.001	0.023		
Effect Size η^2^	0.82	0.83	0.80	0.45		

Abbreviations: LBM = lean body mass; SMM = skeletal muscle mass; $\overset{\cdot }{V}O_{2}$max = maximal oxygen uptake; ^○^ *p* < 0.05, ^○○^ *p* < 0.01—significantly different from the general preparation phase in a controls group; ^++^ *p* < 0.001—significantly different from sprinters at the same training phase; * *p* < 0.05, ** *p* < 0.01, *** *p* < 0.001—significantly different from controls at the same training phase.
